# Identification of genetic linkage group 1-linked sequences in Japanese eel (*Anguilla japonica*) by single chromosome sorting and sequencing

**DOI:** 10.1371/journal.pone.0197040

**Published:** 2018-05-08

**Authors:** Kazumi Matsubara, Yuki Iwasaki, Issei Nishiki, Kazuharu Nomura, Atushi Fujiwara

**Affiliations:** 1 National Research Institute of Fisheries Science, Japan Fisheries Research and Education Agency, Yokohama, Kanagawa, Japan; 2 National Research Institute of Aquaculture, Japan Fisheries Research and Education Agency, Minami-ise, Mie, Japan; National Cheng Kung University, TAIWAN

## Abstract

Japanese eel (*Anguilla japonica*) constitutes one of the most important food fish in Japan; accordingly, genome sequencing and linkage mapping have been conducted for the purpose of artificial cultivation. In the next stage, integration of genomic sequences within linkage groups (LG) is required to construct higher-resolution genetic markers for quantitative trait loci mapping and selective breeding of beneficial traits in farming. In order to identify LG1-linked scaffolds from the draft genome assembly (323,776 scaffolds) reported previously, we attempted to isolate chromosomes corresponding to LG1 by flow sorting and subsequent analyses. Initially, single chromosomes were randomly collected by chromosome sorting and subjected to whole-genome amplification (WGA). A total of 60 WGA samples were screened by PCR with primers for a known LG1-linked scaffold, and five positive WGA samples were sequenced by next-generation sequencing (NGS). Following reference mapping analysis of the NGS reads, four of the five WGA samples were found to be enriched by LG1-linked sequences. These samples were cytogenetically assigned to chromosome 5 by fluorescence *in situ* hybridization. Using blastn searches with 82,081 contigs constructed from the NGS reads of the four WGA samples as queries, 2323 scaffolds were identified as putative LG1-linked scaffolds from the draft genome assembly. The total length of the putative LG1-linked scaffolds was 99.0 Mb, comparable to the estimated DNA amounts of chromosome 5 (91.1 Mb). These results suggest that the methodology developed herein is applicable to isolate specific chromosome DNAs and integrate unanchored scaffold sequences onto a particular LG and chromosome even in teleost fishes, in which isolation of specific chromosomes by flow sorting is generally difficult owing to similar morphologies, sizes, and GC-contents among chromosomes in the genome. The putative LG1-linked scaffolds of Japanese eel contain a total of 6833 short tandem repeats which will be available for higher-resolution linkage mapping.

## Introduction

Japanese eel (*Anguilla japonica*) constitutes one of the most important fish for cultivation in East Asia, and of Japan in particular. However, the population size of this species has declined owing to overfishing and destruction of their natural habitat by humans. This species was accordingly categorized as an endangered species (class EN) by the International Union for Conservation of Nature (IUCN) Red List in June 2014. Artificial cultivation of this species has therefore been attempted with high priority in fisheries to supply stable amounts of the fish for market. Building upon the marked advances in technologies and knowledge of artificial reproduction over the last few decades [1–3], our institute has recently achieved the full-life cycle aquaculture of the Japanese eel under artificial conditions for the first time [4].

Although further improvements are still required for commercial-scale production, it is currently possible to artificially produce progeny for genetic studies and to improve production performance using selective breeding programs. To facilitate this process, we constructed genetic linkage maps [5, 6] and identified 19 linkage groups (LGs) using double digest restriction-site associated DNA sequencing (ddRAD-Seq) [6]. A quantitative trait locus (QTL) significantly associated with the timing of metamorphoses from leptocephali to glass eel, one of the most important traits in farming, was genetically mapped close to a ddRAD tag at the terminal region of LG1 [7]. The first draft genome, having a size of 1.15 Gb and consisting of over 323,776 scaffolds, has also been published for this species [8]. However, although we performed blastn searches with ddRAD tag sequences against the draft genome sequences to anchor scaffolds onto linkage groups, only 1252 scaffolds with a total length of 151 Mb, comparable to 13% of the genome size, were anchored to the LGs [6]. Moreover, the candidate sequence of the QTL for timing of metamorphosis has not yet been detected in the sequences of these scaffolds. Integration of larger genomic sequences onto linkage groups is thus required to develop higher resolution genetic markers for QTL mapping and selective breeding of beneficial traits in farming.

Sequencing technologies, library preparation methods, and computational analyses have rapidly advanced over the past decade. These advancements have contributed to spiraling publication of whole genome sequences or draft genome sequences in both model and non-model organisms (e.g., [9, 10]). However, it remains difficult to reconstruct the complete genome sequence in some species. Teleost fish comprise one such species group owing to the occurrence of whole genome duplication in the common ancestor. Many remnants of the whole genome duplication remain, which causes inaccuracy in assembling the genome sequence in the genomes of teleost fishes [6].

Chromosome sorting is a powerful method for isolation of a particular chromosome from chromosome suspensions prepared with cultured cells [11–13]. In addition, chromosome sequencing using next-generation sequencing technologies has been conducted in various animals and plants as well (e.g., [14–19]). This approach can also be used for DNAs amplified from single copy chromosomes isolated by chromosome sorting [20]. From these previous studies, we conceived the idea that sequencing single chromosomes corresponding to one LG of the Japanese eel might be a powerful and economic approach to link additional scaffolds within the LG and thereby improve the genome sequence information thereof. However, isolation of a particular chromosome by chromosome sorting is considered to be impracticable for most fishes as the morphologies, sizes, and GC-contents are relatively similar among chromosomes in the genome of each species (e.g., [21, 22]). It was therefore necessary to develop a strategy to distinguish the chromosome corresponding to a particular LG from randomly collected single chromosomes.

In this study, we obtained chromosome DNAs from isolated single chromosomes corresponding to LG1 of Japanese eel by a combination of single chromosome sorting and PCR. Then, using fluorescence *in situ* hybridization (FISH), we revealed that LG1 corresponded to chromosome 5, the second largest chromosome pair. Finally, the DNAs from isolated chromosomes were sequenced by high through-put sequencing and contigs assembled from the reads were used for screening large numbers of putative LG1-linked scaffolds.

## Materials and methods

### Animals, DNA extraction, and cell culture

An eel fibroblast line (EE2) was established from an embryo obtained by artificial insemination between adult eels purchased from a commercial farm. The embryo was rinsed with PBS, minced, and implanted in wells of 24-well culture plates containing Leibovitz's L-15 medium (Gibco-Thermo Fisher Scientific, Carlsbad, CA, USA) supplemented with fetal bovine serum (FBS, HyClone-GE Healthcare, Cambridge, UK). Fibroblasts were cultured under the condition of 25°C (current passage 181). Once the fibroblast cells had grown to approximately 80% confluency, they were transferred to T25 flasks and continuously cultured. The cultured fibroblasts (passage 119) were used for chromosome sorting.

Genomic DNAs extracted from the blood of wild-captured adult individuals were used as DNA templates in the PCR amplification of partial DNA fragments of LG1 scaffolds for FISH mapping, described below.

### Ethics statement

This project was conducted in accordance with the Guidelines for Animal Experimentation of the National Research Institute of Aquaculture (NRIA) in Mie, Japan. All animal procedures were approved by the Institutional Animal Care and Use Committee of the NRIA.

### Selection of scaffolds and amplification of partial DNA scaffold fragments

To cytogenetically identify the chromosome corresponding to LG1, we selected three scaffolds previously anchored to LG1 [6], scaffold_127 (KI304514), 10233 (KI314607), and 12248 (KI316617), from the draft genome sequence of Japanese eel ([8]; BioProject accession no. PRJNA158309). Scaffold_12248 and 10233 contained genetic markers located near the QTL, and scaffold_127 was the largest among the scaffolds at the distal position in LG1 (S1 Table). As there is high intraspecific variation of genome sequences in Japanese eel, whole-genome NGS reads obtained from the individual used in our laboratory were mapped to sequences of the three scaffolds, and the scaffold sequences were modified for subsequent experiments with our individual. Partial DNA fragments of the three scaffolds were amplified using PCR with the primer sets specific for each scaffold (S1 Table). PCR was conducted using PrimeSTAR GXL DNA polymerase (TaKaRa, Kusatsu, Japan) in the following condition: 30 cycles of 98°C for 10 s, 60°C for 15 s, and 68°C for 8 min. The PCR products were electrophoresed on 1% agarose gels, extracted from the prominent bands using the Zymoclean™ Large Fragment DNA Recovery Kit (Zymo Research, Irvine, CA, USA), and utilized for FISH.

### Chromosome preparation and FISH

Chromosome slides were prepared from lymphocytes as described by Fujiwara et al. [23]. Mitogen-stimulated lymphocytes were cultured in L-15 medium supplemented with 10% FBS, 18 μg/ml phytohemagglutinin (PHA-W), 100 μg/ml lipopolysaccharide, and 25 μM of 2-mercaptoethanol. Colcemid (KaryoMAX®; Gibco-Thermo Fisher Scientific) was added to the culture flask at 500 ng/ml as the final concentration prior to harvesting. Following harvesting, cultured cells were suspended in 0.075 M KCl and then fixed in 3:1 methanol:acetic acid. The cell suspension was dropped onto glass slides, air-dried, and stored at −80°C. Giemsa-stained chromosome slides were prepared, and relative ratios of each chromosome to the total genome size were measured using ImageJ [24].

FISH was performed as described in our previous study with slight modifications [25]. The mixture of PCR products from one scaffold was labeled with biotin-16-dUTP (Roche, Basel, Switzerland) or digoxigenin-11-dUTP (Roche) using a nick translation kit (Roche). Labeled probes were purified by ethanol precipitation, mixed with hybridization buffer (50% formamide, 2X SSC, 10% dextran sulfate, and 1 mg/ml BSA) and denatured by incubation at 75°C for 10 min. Chromosome slides were denatured in 70% formamide (v/v)/2X SSC for 2 min at 70°C. Approximately 70 ng of labeled probe per slide was hybridized onto metaphase chromosomes for 1 day at 37°C. Post-hybridization washes were carried out as follows: 50% formamide (v/v)/2X SSC for 20 min at 37°C, 2X SSC for 15 min at room temperature, 1X SSC for 15 min at room temperature, and 4X SSC for 5 min at room temperature. The hybridized DNA probes were reacted with streptavidin, Alexa Fluor® 488 conjugate (Molecular Probes-Thermo Fisher Scientific) or anti-digoxigenin rhodamine, Fab fragments (Roche) diluted in 1% BSA/4X SSC for 1 h at 37°C. The chromosome slides were washed as follows: 4X SSC for 5 min at room temperature, 0.1% Nonidet P-40/4X SSC for 5 min at room temperature, and 4X SSC for 5 min at room temperature. Then, the slides were counter-stained with 20 μg/ml DAPI in 2X SSC and mounted with VectaShield (Vector Laboratories, Burligame, CA, USA). Fluorescent images were captured using a Nikon DS-Ri2 digital camera (Nikon, Tokyo, Japan) mounted on a Nikon Eclipse 90i microscope, and analyzed using NIS-Elements AR imaging software (Nikon).

### Chromosome sorting

Chromosome suspension for flow sorting was prepared using the protocol described in Sillar and Young [26]. EE2 cells were cultured in T75 flasks and colcemid was added to the culture flask at 500 ng/ml as the final concentration for 16 h prior to harvesting. Mitotic cells were harvested by vigorous shaking of the culture flask. Following harvesting, the cells were rinsed with PBS and then suspended in 0.075 M KCl for 30 min. After the hypotonic treatment, cells were rinsed with polyamine buffer (PAB: 15 mM Tris-HCl, 2 mM EDTA, 0.5 mM EGTA, 80 mM KCl, 20 mM NaCl, 0.2 mM spermine, 0.5 mM spermidine, and 14 mM of 2-mercaptoethanol) and finally resuspended in 0.1% digitonin in PAB. The cell suspension was intermittently mixed by vortexing for 30–60 s to disrupt the cell membranes. The chromosomes were stained with chromomycin A3 and Höechst33258 prior to flow sorting. The chromosome suspension was analyzed using a MoFlo XDP cell sorter (Beckman Coulter Inc., Brea, CA, USA) and single chromosomes were separately collected into PCR tubes.

### Amplification of chromosome DNA and PCR-screening

Each chromosome DNA obtained via the chromosome sorting step was amplified using a PicoPLEX™ WGA Kit (Rubicon Genomics, Ann Arbor, MI, USA) according to the manufacturer’s protocol. The volume for all reaction steps was scaled down to half.

The amplified chromosome DNAs were screened by PCR with primers designed to amplify a short partial fragment (approximately 150 bp) of scaffold_127: scf_127_short_F (5′-GCT AGT GAT CTT GGG CCC TC-3′) and scf_127_short_R (5′-CAC GAT TTG CCC TGC AGT TC-3′). PCR was conducted using Ex Taq (TaKaRa) in the following condition: an initial denaturation at 94°C for 2 min, followed by 35 cycles of 94°C for 30 s, 65°C for 30 s, and 72°C for 30 s; with 72°C for 5 min for a final extension. The PCR products were electrophoresed on 2% agarose gels. The chromosome DNAs that showed a band of the expected size in gel electrophoresis were used for FISH using the protocol described above, and for high-throughput sequencing.

### High-throughput sequencing

As the size of DNAs amplified by the WGA Kit ranged from 200 to 3000 bp (see Result section and Panel A in S1 Fig), advanced sequencing technologies such as PacBio and Nanopore Sequencing, that can produce long sequence reads, were thought to be unsuitable for high-throughput sequencing of the amplified chromosome DNAs. Therefore, we used Ion Proton platform for the sequencing. The amplified chromosome DNAs that showed positive amplicons in PCR-screening for LG1 was fragmented to lengths of approximately 200-bp using the Covaris S220 system (Woburn, MA, USA). The fragmented DNA was purified using DNA Clean & Concentrator™-5 (Zymo Research), eluted with 10 mM Tris-HCl (pH 8.0), and then ligated to P1 and index adapters using the Ion Plus Fragment Library Kit (Ion Torrent-Thermo Fisher Scientific) according to the manufacturer’s protocol. After adapter ligation, each DNA sample was purified using Agencourt AMPure XP beads (Beckman Coulter Inc.). Fragments were size-selected using BluePippin and a 2% agarose cartridge (Sage Science, Beverly, MA, USA) under a “tight” setting with a mean of 280 bp. After size selection, each DNA sample was purified using Agencourt AMPure XP beads and then amplified using KAPA HiFi polymerase (KAPA Biosystems, Woburn, MA, USA) with Library Amplification Primer Mix (Ion Torrent-Thermo Fisher Scientific). The following PCR protocol was used: initial denaturation at 95°C for 3 min; eight cycles of 98°C for 20 s, 60°C for 15 s, and 72°C for 30 s; followed by a final extension at 72°C for 1 min. The amplified library was purified using Agencourt AMPure XP beads and quantified using the Qubit fluorometer with a Quant-it dsDNA HS kit (Invitrogen-Thermo Fisher Scientific). The quality and size were finally checked using the Agilent 2200 TapeStation (Agilent Technologies, Santa Clara, CA, USA). Emulsion PCR and Ion Sphere Particle enrichment were carried out using the Ion OneTouch 2 system with an Ion P1 Template OT2 200 kit v3 (Ion Torrent-Thermo Fisher Scientific). Template samples were sequenced on Ion P1 Chip v2 using the Ion Proton platform with an Ion P1 Sequencing 200 kit v3 (Ion Torrent-Thermo Fisher Scientific). Data from the Ion Proton runs were processed using Ion Torrent Suite 4.2.1 software to generate sequenced reads.

### Sequence data analyses

The reads generated by high-throughput sequencing were filtered based on quality using the Trim Sequences program in CLC Genomics Workbench (CLC Bio, Aarhus, Denmark), with Discard reads below length set at 150. Then, the filtered reads were mapped to Japanese eel scaffolds that were anchored onto each LG (1–19 and unanchored scaffolds) in our previous study (Kai et al. 2014), using CLC Genomics Workbench with the following parameters: mismatch cost of 2, insertion cost of 3, deletion cost of 3, length fraction of 0.95, similarity of 0.9, and non-specific match handling of ignore. The number of mapped reads and coverage to each LG were computed using CLC Genomics Workbench. When reads from a chromosome DNA sample were exclusively mapped to LG1, the sample was identified as a derivative from a single chromosome corresponding to LG1. The reads from the correct chromosome DNA sample were assembled using Newbler 3.0 (Roche Diagnostics, Basel, Switzerland) with default parameters.

To screen putative LG1-linked scaffolds, blastn searches were carried out using the assembled contigs against the Japanese eel scaffolds with e-value lower than 1e−20. In cases where the query hit two or more loci with a less than two-fold difference in the bit score, assignments were not made. Scaffolds that aligned with two or more contigs were recognized as putative LG1-linked scaffolds. Then, di—hexanucleotide simple tandem repeats (STR) were searched for the putative LG1-linked scaffolds using Tandem Repeats Finder (ver 4.09) with default parameters.

## Results

### Identification of a chromosome corresponding to LG1

**Fig 1:**
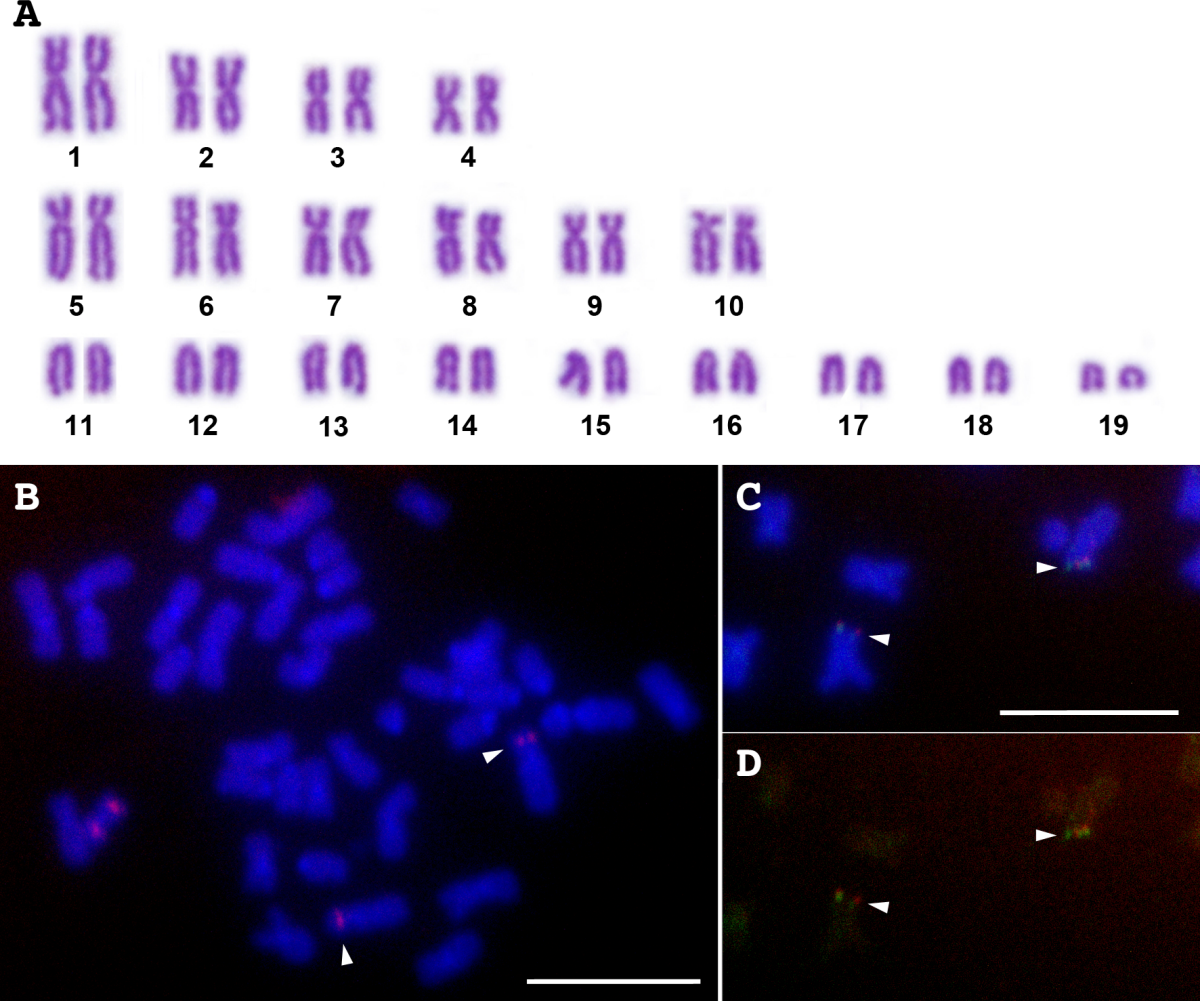
Karyotype and FISH mapping probed with PCR amplicons of LG1-linked scaffolds in Japanese eel. Giemsa-stained karyotype of a wild-captured Japanese eel (A). Hybridization of rhodamine-labeled scaffold_127 amplicons (red signals) on a DAPI-stained metaphase spread (B). Hybridization of Alexa Fluor® 488-labeled scaffold_10233 (green signals) and rhodamine-labeled 12248 (red signals) amplicons on a DAPI-stained metaphase spread (C), and image of fluorescent signals only on the same metaphase spread (D). Arrowheads indicate hybridization signals. Scale bars represent 10 μm.

The chromosome number of Japanese eel is 2n = 38, which consists of 4 pairs of metacentric chromosomes, 6 pairs of submetacentric chromosomes, and 9 pairs of acrocentric chromosomes (Fig 1A). Relative lengths of each chromosome range from 9.0% for the largest chromosome pair, chromosome 1, to 2.3% for the smallest chromosome pair, chromosome 19 (S2 Table). The mixture of PCR products from scaffold_127 was mapped to central region of the short arm of chromosome 5, the second largest chromosome (Fig 1B). Both scaffold_10233 and 12248 were mapped to the telomeric region of chromosome 5 (Fig 1C and 1D). This result is consistent with the result of linkage mapping in which the two markers, Ajp-s3698 and Ajp-s1912, respectively mapped to the most distal and the second distal position in LG1 [6] (S1 Table). The FISH data did not show sufficient resolution for the orientation between the two scaffolds to be ascertained (Fig 1D).

### Collection of single chromosomes and screening of chromosome 5

**Fig 2:**
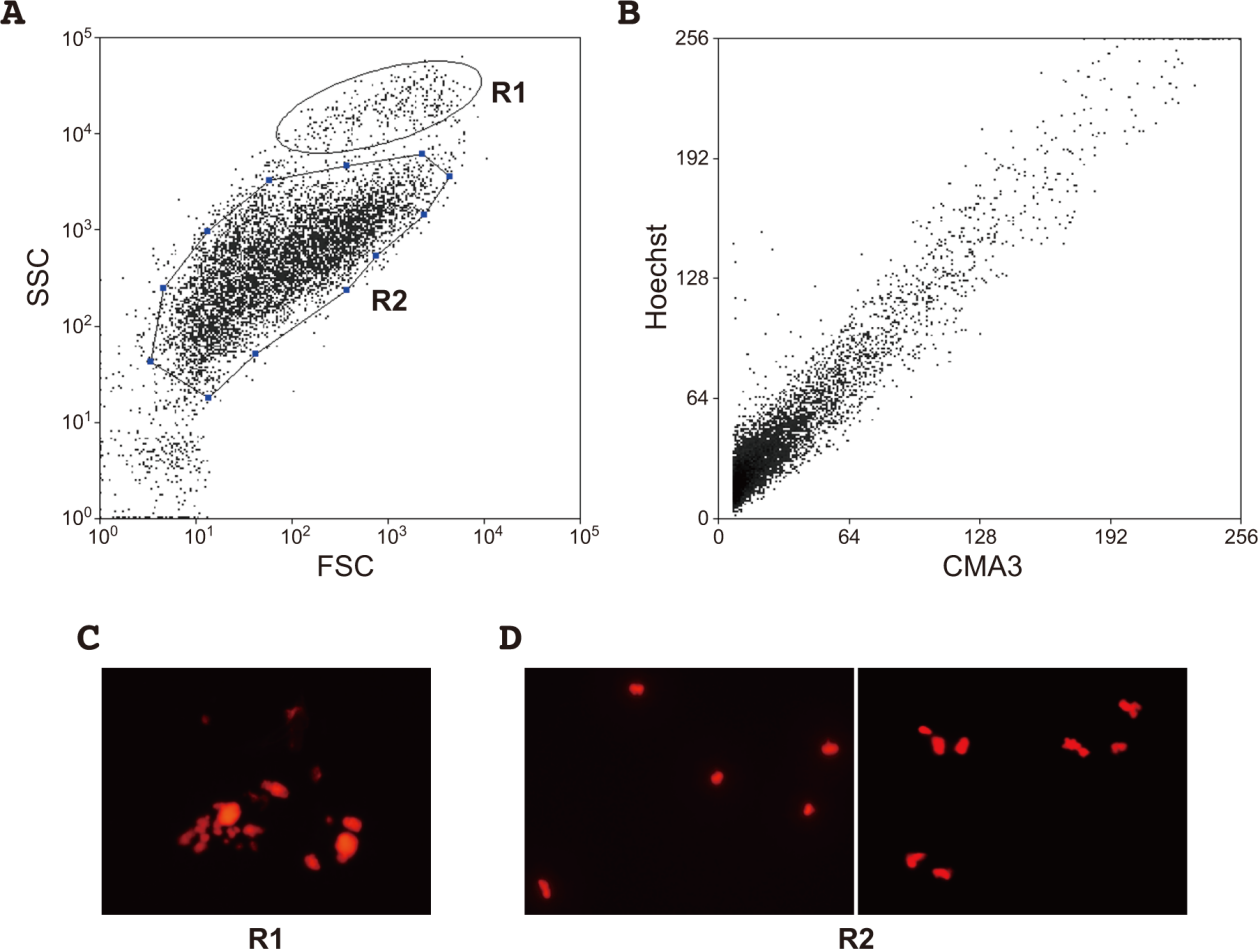
Chromosome sorting in Japanese eel. Bivalent profile of flow sorting with forward scatter (FSC) and side scatter (SSC) is shown (A). Bivalent profile of flow sorting with chromomycin A3 (CMA3) and Hoechst33258 (Hoechst) for samples that pass region 2 (R2) is shown (B). Droplets that pass region 1 (R1) and R2 of (A) contained debris of nuclei and dividing cells (C), and chromosomes (D), respectively.

Initially, we analyzed a bivariate sorting profile with parameters for size and shape of samples in droplets. The profile presented compartmentalization with two clusters of plots, conveniently termed region 1 (R1) and 2 (R2) (Fig 2A). The objects in R1 comprised large debris and clumps of chromosomes that were likely generated through preparation of the chromosome suspension (Fig 2C). The R2 objects were enriched with single chromosomes (Fig 2D). Next, a bivariate sorting profile were analyzed using parameters for two fluorescent dyes, chromomycin A3 and Höechst33258, for samples contained in R2 (Fig 2B). The profiles showed no clear compartmentalization of plots (Fig 2B). Therefore, single chromosomes were separately sorted from the samples of R2.

Chromosome DNAs were amplified from a total of 60 sorted samples. The amplicons ranged from 200 to 3000 bp (e.g., Panel A in S1 Fig). PCR with the primer set for detection of scaffold_127 gave rise to a prominent band with the expected size (approximately 150 bp) in five of the 60 samples, EE2-sc-5, 6, 7, 8, and 15 (Panel B in S1 Fig).

### High-throughput sequencing and read mapping

High-throughput sequencing with the Ion Proton platform yielded 2,298,094, 2,436,681, 7,486,473, 3,339,488, and 1,731,200 reads for EE2-sc-5, 6, 7, 8, and 15, respectively (S3 Table). After removing low quality reads, the following numbers of reads were retained: 1,495,839 (mean length 206.8 bp) for EE2-sc-5, 1,600,331 (mean length 206.7 bp) for EE2-sc-6, 4,952,072 (mean length 206.7 bp) for EE2-sc-7, 2,174,130 (mean length 207.3 bp) for EE2-sc-8, and 1,118,036 (mean length 206.4 bp) for EE2-sc-15 (S3 Table). Of 840,736 total mapped reads from the EE2-sc-5, 126,951 (15.10%) were mapped to the reference genome sequences of LG1 (Table 1). The total length of the reads mapped to LG1 showed 2.43-fold coverage of the reference sequence of LG1 (S4 Table). A total of 2827 reads corresponding to 0.34% of all mapped reads were mapped to the other LGs (Table 1). The remaining reads, i.e. 710,958 (84.56%), were mapped to the unanchored scaffolds. In a similar manner, approximately 13% of total mapped reads were mapped to the LG1 reference sequences in EE2-sc-6, 8, and 15 (Table 1), and consequently yielded 1.54–3.09-fold coverage of the reference sequences (S4 Table). Moreover, the majority of reads (over 85% of the total) were mapped to the unanchored scaffolds in EE2-sc-6, 8, and 15 (Table 1). These results were considered reasonable as only 13% of the draft genome sequences were anchored to the LGs in our previous study [6]. In comparison, 150,993 (5.53% of the total mapped reads), 53,333 (1.95%), and 131,040 (4.80%) reads of EE2-sc-7 were mapped to the reference sequences of LG1, 15, and 16, respectively (Tables 1 and S4). The total lengths of the reads mapped to respective LGs exhibited 2.88 (LG1), 2.34 (LG15), and 4.25-fold (LG16) coverage of the reference sequence (S4 Table). It was thus likely that the DNA sample of EE2-sc-7 consisted of amplicons from three chromosomes corresponding to LG1, 15, and 16.

**Table 1:** Result of read mapping to the reference genome sequences of Japanese eel.

Sample ID	Total mapped reads	Mapped reads (mapped/total %)^a^		
		LG1	LG2–19	Others
EE2-sc-5	840,736	126,951	2,827	710,958
		(15.10%)	(0.34%)	(84.56%)
EE2-sc-6	880,857	116,595	2,524	761,738
		(13.24%)	(0.29%)	(86.48%)
EE2-sc-7	2,731,576	150,993	199,980	2,380,603
		(5.53%)	(7.32%)	(87.15%)
EE2-sc-8	1,175,039	161,522	4,019	1,009,498
		(13.75%)	(0.34%)	(85.91%)
EE2-sc-15	600,306	80,744	1,868	517,694
		(13.45%)	(0.31%)	(86.24%)

^a^Scaffold sequences of the Japanese eel draft genome [8] were anchored to LGs in our previous study [6]. The scaffolds that were not anchored to any LGs were categorized into "Others".

### Chromosome painting

**Fig 3:**
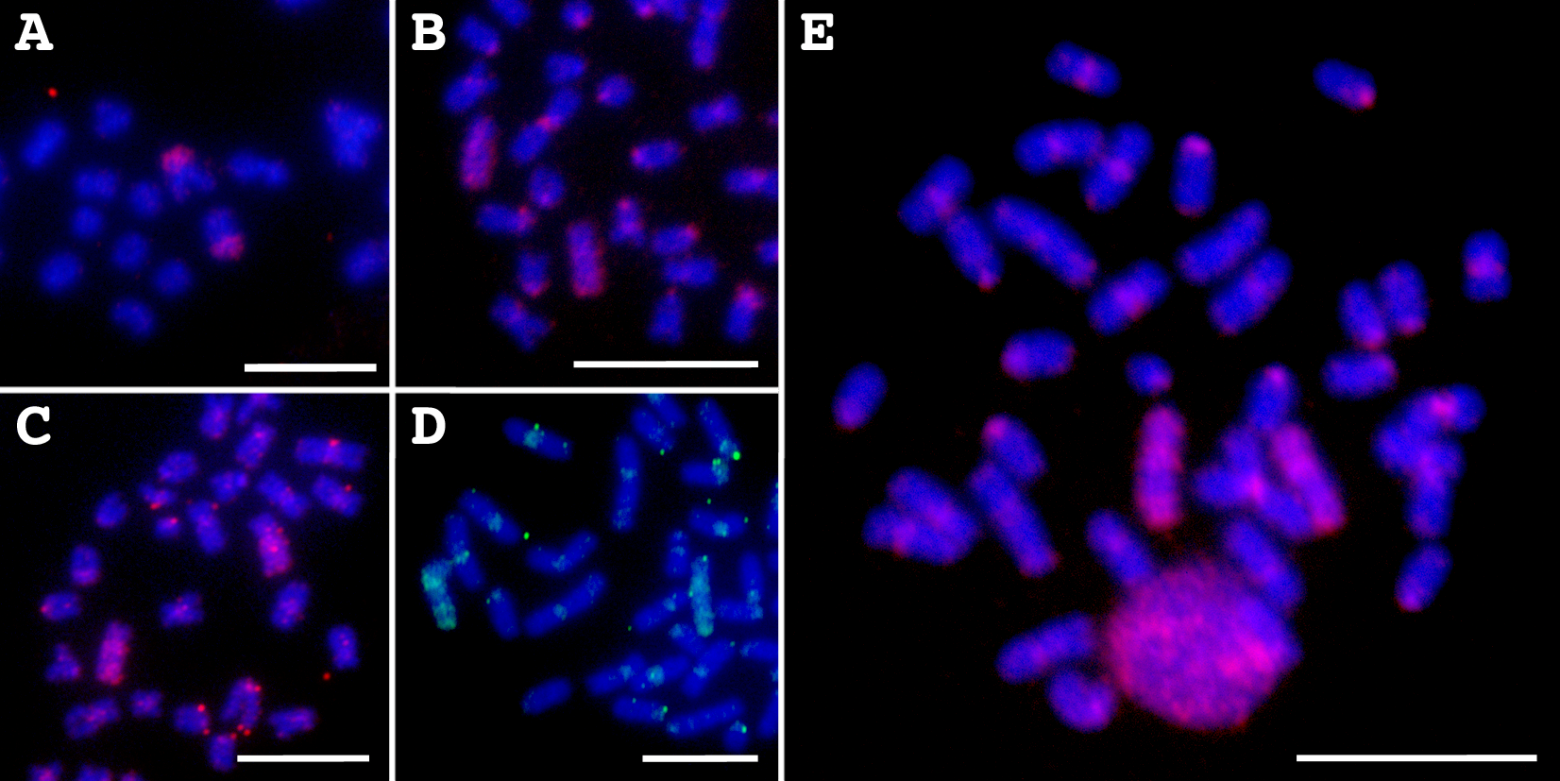
Chromosome painting with WGA products from single chromosomes. Chromosome painting probed with WGA products from sorted single chromosome samples EE2-sc-5sc (A), 6 (B), 8 (C), and 15 (D) on metaphase spreads from a Japanese eel cell line (EE2). The probes were labeled with rhodamine (red signals; A-C) or Alexa Fluor® 488 (green signals; D). Chromosome painting probed with a mixture of the four products on a metaphase spread of a wild-captured animal (E). Scale bars represent 10 μm.

In FISH analyses, amplified chromosome DNAs from EE2-sc-5 were hybridized to the short arm of chromosome 5 but not to the long arm (Fig 3A), indicating that the DNA was derived from a fragment of chromosome 5. Chromosome DNAs of EE2-sc-6, 8, and 15 were hybridized to whole regions of chromosome 5 (Fig 3B–3D). All of the three chromosome DNAs produced hybridization signals on centromeric regions of all chromosomes. These results indicate that the three chromosome DNAs were derived from whole single chromosome 5. A DNA mixture of the four chromosome DNAs hybridized to the whole regions of chromosome 5 with intense fluorescent signals (Fig 3E).

### Assembly of sequence reads from chromosome DNA and screening of putative LG1-linked scaffolds

Assembly of a total of 6,388,336 reads from the four chromosome samples (EE2-sc-5, 6, 8, and 15) yielded 82,081 contigs with a mean length of 434.2 bp (maximum 3126 bp) (Accession no. BEYV01000000). By blastn search with the contigs, 2323 scaffolds were screened from Japanese eel genome data (KI304388–KI391930) as candidates for LG1-linked scaffolds (S5 Table). The total length of the candidate scaffolds selected in the present studies was 99.0 Mb. Of 88 scaffolds anchored to LG1 in our previous study [6], 82 were selected as candidates again in this study. However, the candidates also included 24 scaffolds previously anchored to LGs other than LG1 [6] (S5 Table). The total length of the 24 scaffolds was 7.9 Mb. The remaining 2217 scaffolds were not anchored to any LGs in our previous study [6] (S5 Table). Of the 2323 putative LG1-linked scaffolds, 1486 contained STRs; the number of STRs in scaffolds ranged from 1 to 63 (S5 Table). The total number of STRs from the all putative LG1-linked scaffolds was 6833: 3633 of di-, 999 of tri-, 1217 of tetra-, 355 of penta-, and 629 of hexanucleotide repeats.

**Fig 4:**
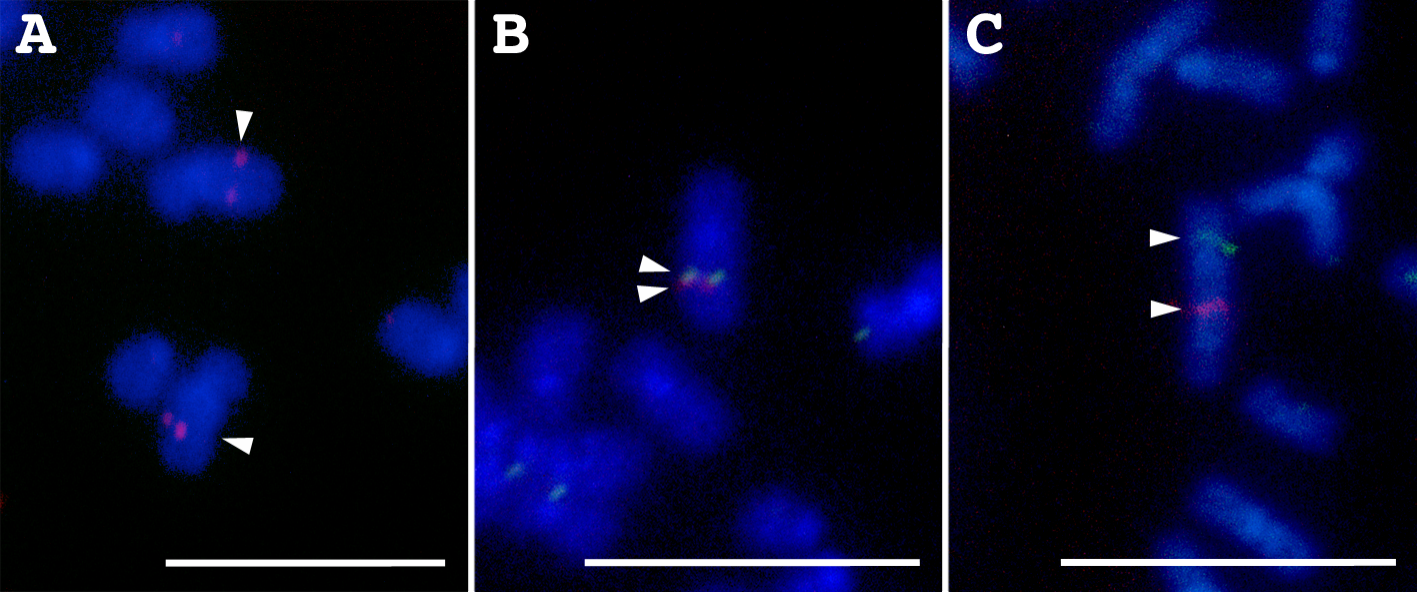
FISH mapping with putative LG1-linked scaffolds in Japanese eel. PCR amplicons from scaffold_12 (A), 135 and 297 (B), and 256 and 997 (C) are mapped to chromosome 5. The amplicons of scaffold_12 (A), 135 (B), and 256 (C) were labeled with rhodamine (red signals), and scaffold_297 (B) and 997 (C) were labeled with Alexa Fluor® 488 (green signals). Arrowheads indicate hybridization signals. Scale bars represent 10 μm.

Partial fragments were amplified by long PCR from five candidate LG1-linked scaffolds (scaffold_12, 135, 256, 297, and 997), which were not previously anchored to any LGs [6], and then used for FISH to confirm their chromosome locations (see S6 Table for PCR primers). All the amplicons from the five scaffolds were mapped to chromosome 5; scaffold_997 to a proximal region on 5p, 256 to a proximal region on 5q, and the remaining three to central regions on 5q (Fig 4).

## Discussion

### Validation of the approach for collecting single chromosome DNA by chromosome sorting

In this study, we aimed to develop new approach with single chromosome sorting to screen the scaffolds which linked to LG1 from the Japanese eel draft genomes comprising of relatively short scaffolds.

In chromosome sorting for mammals such as human and mouse, compartmentalization of spots by a particular chromosome was clearly observed on profile graphs (e.g., [13, 27, 28]). In contrast, it has been considered that isolation of particular chromosomes is generally difficult for most fishes because the chromosome morphologies, sizes, and GC-contents are relatively similar among chromosomes in the genomes of respective fish species (e.g., [21, 22]). Moreover, the use of cell culture to obtain vast number of dividing cells is also laborious in fishes. In the present study, we established a cell line from Japanese eel and applied chromosome sorting to isolate chromosomes in a fish for the first time. There was no obvious compartmentalization of the spots on profiles in chromosome sorting (Fig 2), as would be expected to be case for most fishes. However, we successfully analyzed chromosome DNAs corresponding to LG1 by well-suited screening steps as itemized below.

First, we demonstrated by FISH mapping of partial DNA segments of known LG1-linked scaffolds, that LG1 corresponds to chromosome 5. Then, five chromosome DNA samples of LG1 were screened from a total of 60 samples through chromosome sorting and PCR testing. Read mapping to known scaffolds revealed that one of the five samples (EE2-sc-7) comprised a mixture of chromosome DNAs from three LGs (LG1, 14, and 15), likely owing to adhesion of chromosomes in the chromosome suspension or contamination at sorting. Chromosome painting showed that three (EE2-sc-6, 8, and 15) of the other four samples and the remaining sample (EE2-sc-5) were respectively derived from single chromosomes and a chromosome fragment of chromosome 5. As the haploid number of Japanese eel is 19, it was expected that one chromosome 5 should be contained among 19 chromosome samples randomly collected by chromosome sorting (probability 1/19 ≈ 5.26%). Thus, we successfully obtained single chromosome 5 or a fragment of chromosome 5 with a reasonable probability (4/60 ≈ 6.67%). Our study therefore provides an organized strategy to obtain pure DNA from single chromosomes in fishes through a combination of chromosome sorting, PCR screening, and cytogenetic and genomic analyses.

### Effective screening of the LG1-linked scaffolds

The relative ratio of chromosome 5 (LG1) is 7.92% in the genome of Japanese eel (S2 Table). The estimated genome size is 1.15 Gb [8], indicating that the estimated physical size of chromosome 5 is approximately 91.1 Mb (S2 Table). The blastn search with contigs from reads of four DNA samples of chromosome 5 against the Japanese eel draft genome sequence screened 2323 scaffolds as being putatively LG1-linked with a total scaffold length of 99.0 Mb, which was close to the estimated size of chromosome 5. All five tested putative LG1-linked scaffolds were mapped to chromosome 5. Thus, it is expected that the putative LG1-linked scaffolds selected in this study are actually located on chromosome 5 with high probabilities. Although physical mapping will be necessary to identify the exact locations of the scaffolds, the screened scaffolds are available for comparison of chromosome homologies with other species and for development of new genetic markers for precise QTL mapping. A total of 6833 STRs was detected in the putative LG1-linked scaffolds. These STR sites will be useful for designing high-density genetic markers for QTL mapping and selective breeding of beneficial traits in farming.

In general, there has been two ways to link scaffold sequences onto linkage groups/chromosomes. One is to utilize single molecule sequencing technologies such as PacBio Sequencing System and Nanopore Technologies that produce long continuous reads. The other one is linkage mapping in which linkage markers are available for blast search to draft genomes as shown in our previous study [6]. The methodology developed in this study has the potential as a new option.

### Application of the methodology

In this study, we began screening single chromosomes corresponding to LG1 based on the sequence information of three known LG1-linked scaffolds (scaffold_127, 10233, and 12248), and finally identified many putative LG1-linked scaffolds using the sequence data from the screened single chromosomes. We previously anchored more than 20 scaffolds to each LG by blastn search with ddRAD tag sequences [6]. It should, therefore, be possible to screen chromosomes corresponding to each LG and identify putative scaffolds linked to the LG using the methodology developed here. As described in the Introduction, teleost fishes are species in which assembling genome sequences with high qualities is difficult owing to the teleost-specific whole genome duplication. Isolating specific chromosome based on the chromosome morphologies and GC contents is also nearly impossible. The combination of a standard genome sequencing approach and the single chromosome sequencing approach developed here will thus constitute a powerful tool for building up whole genome sequences in most fishes. Our methodology will also likely be applicable to any species whose tissues are available for cell culture. For example, although the whole genome sequences are published for some birds and reptiles, the genome sequencing has not been completed for their microchromosomes. Our methodology will be available to screen the scaffolds linked to specific microchromosomes in these organisms. It will be also possible to screen the sequences linked to less-differentiated sex chromosomes such as occur in several vertebrates such as paleognath birds, henophidian snakes, and many amphibians and fishes as well.

## Supporting information

S1 Fig Gel electrophoreses images for WGA products from sorted chromosomes and PCR testing. Amplicons from sorted chromosomes were electrophoresed on 1% agarose gels (a). PCR products amplified using primer sets for a partial fragment of scaffold 127 were electrophoresed on 2% agarose gels (b). M, marker.
(PDF)

S1 Table Scaffolds and primers used for cytogenetic identification of Japanese eel LG1. (XLSX)

S2 Table Relative lengths and estimated physical sizes of each chromosome. (XLSX)

S3 Table Summary of NGS analyses. (XLSX)

S4 Table Results of read mapping to the reference genome sequences of Japanese eel. (XLSX)

S5 Table List of scaffolds screened through blastn searches using chromosome 5-derived contigs. (XLSX)

S6 Table Primers for PCR amplification of the partial fragments of putative LG1-linked scaffolds in Japanese eel used for FISH mapping. (XLSX)

## References

[1] Tanaka H, Kagawa H, Ohta H, Unuma T, Nomura K. The first production of glass eel in captivity: fish reproductive physiology facilitates great progress in aquaculture. Fish Physiol Biochem. 2003; 28(1–4): 493–497. doi: 10.1023/B:FISH.0000030638.56031.ed

[2] Okamura A, Horie N, Mikawa N, Yamada Y, Tsukamoto K. Recent advances in artificial production of glass eels for conservation of anguillid eel populations. Ecol Freshw Fish. 2014; 23(1): 95–110. doi: 10.1111/eff.12086

[3] Tanaka H. Progression in artificial seedling production of Japanese eel *Anguilla japonica*. Fish Sci. 2015;81: 11–19. doi: 10.1007/s12562-014-0821-z

[4] Masuda Y, Imaizumi H, Oda K, Hashimoto H, Teruya K, Usuki H. Japanese eel *Anguilla japonica* larvae can metamorphose into glass eel within 131 days after hatching in captivity. Nippon Suisan Gakkaishi. 2011; 77(3): 416–418.

[5] Nomura K, Ozaki A, Morishima K, Yoshikawa Y, Tanaka H, Unuma T, et al A genetic linkage map of the Japanese eel (*Anguilla japonica*) based on AFLP and microsatellite markers. Aquaculture. 2011; 310(3–4): 329–342.

[6] Kai W, Nomura K, Fujiwara A, Nakamura Y, Yasuike M, Ojima N, et al A ddRAD-based genetic map and its integration with the genome assembly of Japanese eel (*Anguilla japonica*) provides insights into genome evolution after the teleost-specific genome duplication. BMC Genomics. 2014; 15: 233 doi: 10.1186/1471-2164-15-233 24669946 10.1186/1471-2164-15-233 PMC3986909

[7] Nomura K, Fujiwara A, Kai W, Ozaki A, Iwasaki Y, Nakamura Y, et al Mapping of quantitative trait loci (QTL) associated with timing of metamorphosis from leptocephali to glass eels in Japanese eel (*Anguilla japonica*). Plant and Animal Genome Conference XXIV (2016). Available from: https://pag.confex.com/pag/xxiv/webprogram/Paper20651.html

[8] Henkel CV, Dirks RP, de Wijze DL, Minegishi Y, Aoyama J, Jansen HJ, et al First draft genome sequence of the Japanese eel, *Anguilla japonica*. Gene. 2012; 511(2): 195–201. doi: 10.1016/j.gene.2012.09.064 23026207 10.1016/j.gene.2012.09.064

[9] Genome 10K Community of Scientists. Genome 10K: a proposal to obtain whole-genome sequence for 10,000 vertebrate species. J Hered. 2009; 100(6): 659–674. doi: 10.1093/jhered/esp086 19892720 10.1093/jhered/esp086 PMC2877544

[10] Koepfli KP, Paten B, Genome 10K Community of Scientists, O'Brien SJ. The Genome 10K Project: a way forward. Annu Rev Anim Biosci. 2015; 3: 57–111. doi: 10.1146/annurev-animal-090414-014900 25689317 10.1146/annurev-animal-090414-014900 PMC5837290

[11] Lebo RV, Carrano AV, Burkhart-Schultz K, Dozy AM, Yu LC, Kan YW. Assignment of human β-, γ-, and δ-globin genes to the short arm of chromosome 11 by chromosome sorting and DNA restriction enzyme analysis. Proc Natl Acad Sci USA. 1979; 76(11): 5804–5808. 293684 10.1073/pnas.76.11.5804 PMC411739

[12] Lebo RV. Chromosome sorting and DNA sequence localization. Cytometry. 1982; 3(3): 145–154. doi: 10.1002/cyto.990030302 6293786 10.1002/cyto.990030302

[13] Fantes JA, Green DK. Human chromosome analysis and sorting. Methods Mol Biol. 1990; 5: 529–542. doi: 10.1385/0-89603-150-0:529 21374148 10.1385/0-89603-150-0:529

[14] Chen W, Kalscheuer V, Tzschach A, Menzel C, Ullmann R, Schulz MH, et al Mapping translocation breakpoints by next-generation sequencing. Genome Res. 2008; 18(7): 1143–1149. doi: 10.1101/gr.076166.108 18326688 10.1101/gr.076166.108 PMC2493403

[15] Mayer KF, Taudien S, Martis M, Simková H, Suchánková P, Gundlach H, et al Gene content and virtual gene order of barley chromosome 1H. Plant Physiol. 2009; 151(2): 496–505. doi: 10.1104/pp.109.142612 19692534 10.1104/pp.109.142612 PMC2754631

[16] Sudbery I, Stalker J, Simpson JT, Keane T, Rust AG, Hurles ME, et al Deep short-read sequencing of chromosome 17 from the mouse strains A/J and CAST/Ei identifies significant germline variation and candidate genes that regulate liver triglyceride levels. Genome Biol. 2009; 10(10): R112 doi: 10.1186/gb-2009-10-10-r112 19825173 10.1186/gb-2009-10-10-r112 PMC2784327

[17] Berkman PJ, Skarshewski A, Lorenc MT, Lai K, Duran C, Ling EY, et al Sequencing and assembly of low copy and genic regions of isolated *Triticum aestivum* chromosome arm 7DS. Plant Biotechnol J. 2011; 9(7): 768–775. doi: 10.1111/j.1467-7652.2010.00587.x 21356002 10.1111/j.1467-7652.2010.00587.x

[18] Vitulo N, Albiero A, Forcato C, Campagna D, Dal Pero F, Bagnaresi P, et al First survey of the wheat chromosome 5A composition through a next generation sequencing approach. PLoS One. 2011; 6(10): e26421 doi: 10.1371/journal.pone.0026421 22028874 10.1371/journal.pone.0026421 PMC3196578

[19] Fluch S, Kopecky D, Burg K, Šimková H, Taudien S, Petzold A, et al Sequence composition and gene content of the short arm of rye (*Secale cereale*) chromosome 1. PLoS One. 2012; 7(2): e30784. 10.1371/journal.pone.0030784 PMC3273464 22328922

[20] Yang H, Chen X, Wong WH. Completely phased genome sequencing through chromosome sorting. Proc Natl Acad Sci USA. 2011; 108(1): 12–17. doi: 10.1073/pnas.1016725108 21169219 10.1073/pnas.1016725108 PMC3017199

[21] Fujiwara A, Fujiwara M, Nishida-Umehara C, Abe S, Masaoka T. Characterization of Japanese flounder karyotype by chromosome bandings and fluorescence in situ hybridization with DNA markers. Genetica. 2007; 131(3): 267–274. doi: 10.1007/s10709-006-9136-z 17273899 10.1007/s10709-006-9136-z

[22] Pinthong K, Gomontean B, Kongim B, Khakhong S, Sriveerachai T, Supiwong W. Cytogenetic comparison and chromosome localization of the nucleolar organizer region of four grouper genera (Pisces, Epinephelinae) from Thailand. Cytologia, 2013; 78(3): 223–234.

[23] Fujiwara A, Nishida-Umehara C, Sakamoto T, Okamoto N, Nakayama I, Abe S. Improved fish lymphocyte culture for chromosome preparation. Genetica. 2001; 111(1–3): 77–89. 11841191 10.1023/a:1013788626712

[24] Schneider CA, Rasband WS, Eliceiri KW. NIH Image to ImageJ: 25 years of image analysis. Nat Methods. 2012; 9(7): 671–675. 22930834 10.1038/nmeth.2089 PMC5554542

[25] Fujiwara A, Abe S, Yamaha E, Yamazaki F, Yoshida MC. Uniparental chromosome elimination in the early embryogenesis of the inviable salmonid hybrids between masu salmon female and rainbow trout male. Chromosoma. 1997; 106(1): 44–52. 9169586 10.1007/s004120050223

[26] Sillar R, Young BD. A new method for the preparation of metaphase chromosomes for flow analysis. J Histochem Cytochem. 1981; 29(1): 74–78. doi: 10.1177/29.1.6162882 6162882 10.1177/29.1.6162882

[27] Rabbitts P, Impey H, Heppell-Parton A, Langford C, Tease C, Lowe N, et al Chromosome specific paints from a high resolution flow karyotype of the mouse. Nat Genet. 1995; 9(4): 369–375. doi: 10.1038/ng0495-369 7795642 10.1038/ng0495-369

[28] Yang F, Alkalaeva EZ, Perelman PL, Pardini AT, Harrison WR, O'Brien PC, et al Reciprocal chromosome painting among human, aardvark, and elephant (superorder Afrotheria) reveals the likely eutherian ancestral karyotype. Proc Natl Acad Sci USA. 2003; 100(3): 1062–1066. doi: 10.1073/pnas.0335540100 12552116 10.1073/pnas.0335540100 PMC298726

