# Supplementary figures and images for: Identification of genetic linkage group 1-linked sequences in Japanese eel (Anguilla japonica) by single chromosome sorting and sequencing
Source: PLoS One. 2018 May 8;13(5):e0197040. doi: 10.1371/journal.pone.0197040 (PMC5940218; doi:10.1371/journal.pone.0197040)

**A**

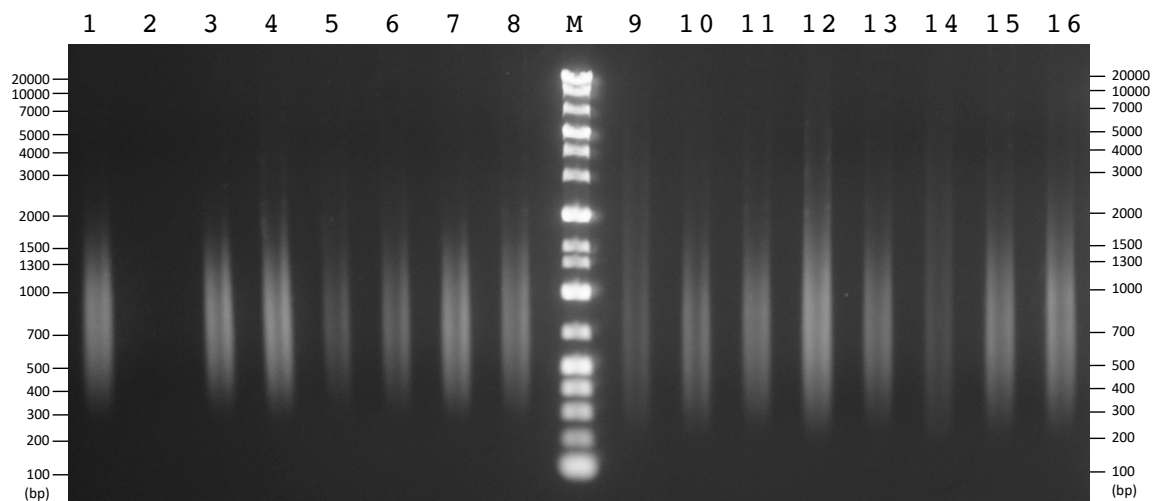

**B**

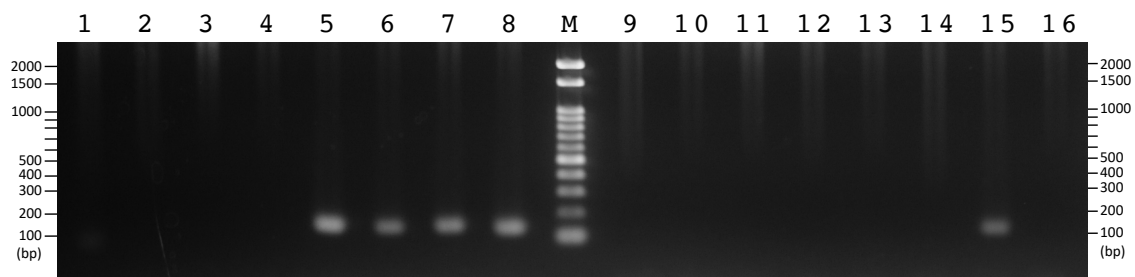

Supplement: S1 Fig — Amplicons from sorted chromosomes were electrophoresed on 1% agarose gels (a). PCR products amplified using primer sets for a partial fragment of scaffold 127 were electrophoresed on 2% agarose gels (b). M, marker. (PDF) [file pone.0197040.s001.pdf]
